# Autoantibodies against islet cell antigens in children with type 1 diabetes mellitus

**DOI:** 10.18632/oncotarget.24527

**Published:** 2018-02-19

**Authors:** Bi-Wen Cheng, Fu-Sung Lo, An-Mei Wang, Chen-Mei Hung, Chi-Yu Huang, Wei-Hsin Ting, Mei-Ore Yang, Chao-Hsu Lin, Chia-Ching Chen, Chiung-Ling Lin, Yi-Lei Wu, Yann-Jinn Lee

**Affiliations:** ^1^ Department of Pediatric Endocrinology, MacKay Children's Hospital, Taipei, Taiwan; ^2^ Department of Pediatrics, Chang Gung Memorial Hospital, Taoyuan, Taiwan; ^3^ College of Medicine, Chang Gung University, Taoyuan, Taiwan; ^4^ Department of Nuclear Medicine, MacKay Memorial Hospital, Taipei, Taiwan; ^5^ Department of Pediatrics, Hsinchu Cathay General Hospital, HsinChu, Taiwan; ^6^ MacKay Medicine, Nursing and Management College, New Taipei City, Taiwan; ^7^ Department of Laboratory Medicine, MacKay Memorial Hospital, Taipei, Taiwan; ^8^ Department of Pediatrics, MacKay Memorial Hospital HsinChu Branch, HsinChu, Taiwan; ^9^ Department of Pediatrics, Chiayi Christian Hospital, Chiayi, Taiwan; ^10^ Department of Medical Research, MacKay Memorial Hospital, Taipei, Taiwan; ^11^ Department of Pediatric Endocrinology and Metabolism, Chuanghua Christian Children's Hospital, Chuanghua, Taiwan; ^12^ Institute of Biomedical Sciences, Mackay Medical College, New Taipei City, Taiwan; ^13^ Department of Medicine, Mackay Medical College, New Taipei City, Taiwan; ^14^ Department of Pediatrics, School of Medicine, College of Medicine, Taipei Medical University, Taipei, Taiwan

**Keywords:** autoantibodies, glutamic acid decarboxylase 65 (GAD) autoantibody, insulinoma-associated protein 2 autoantibody, insulin autoantibody, type 1 diabetes mellitus, Immunology

## Abstract

We investigated the prevalence of glutamic acid decarboxylase 65 autoantibody (GADA), insulinoma-associated protein 2 autoantibody (IA2A), and insulin autoantibody (IAA) in 750 children with type 1 diabetes (T1D) living in Taiwan. GADA, IA2A, and IAA were measured by radioimmunoassay. The data were assessed by *χ*^2^ test, binary logistic regression, and Spearman rank correlation. Of the 750 T1D patients, 66.3% had GADA, 65.3% IA2A, 35.7% IAA, and 17.2% no autoantibodies. The prevalence of GADA and IA2A significantly decreased along T1D duration. The positivity of either GADA or IA2A was 89.4% within the first year of disease and decreased to 36.7% after 9 years (*P* = 1.22 × 10^–20^). Female patients had significantly higher prevalence of GADA compared with male patients (72.3% vs. 59.7%, *P* = 0.00027). The patients diagnosed before 12 years of age had a positive rate of 92.2% for either GADA or IA2A. Patients diagnosed at age 12 or above had a significantly lower positive rate of 81.6% (*P* = 0.011). GADA and IA2A significantly correlated with each other (rs = 0.245, *P* = 1.09 × 10^–11^). We concluded that autoantibodies were detectable in 89.4% of T1D patients within one year after diagnosis. Their prevalence declined with disease duration. GADA was more prevalent in female patients. GADA and IA2A weakly correlated with each other.

## INTRODUCTION

Diabetes mellitus is a chronic metabolic disease with hyperglycemia resulting from insulin deficiency, impaired insulin action, or both [1]. There are various types of diabetes by the etiology. Type 1 diabetes (T1D) is caused by an autoimmune or idiopathic process resulting in the destruction of pancreatic β cells [2]. In Taiwan, the incidence rates of T1D and type 2 diabetes (T2D) are respectively 5.3/100,000 [3] and 6.5/100,000 [4, 5] in children and adolescents. Differentiation between T1D and other types of diabetes can usually be made on clinical manifestations, age at diagnosis, and family history; however, it may be difficult in some cases due to the increasing incidence of T2D in children over the past two decades [6]. Additionally T1D differs from T2D in the clinical and public health burden, disease management, and the risks of acute and chronic complications, therefore clear differentiation between them is imperative for appropriate therapy [7]. Islet autoantibodies are recognized and becoming increasingly important in differentiating various types of diabetes [8]. Among these autoantibodies, glutamic acid decarboxylase 65 autoantibody (GADA), insulinoma-associated protein 2 autoantibody (IA2A), and insulin autoantibody (IAA) are commonly tested.

Although several studies about the above-mentioned autoantibodies in Asians have been reported, however, they were of small numbers of subjects [9, 10] except for only a few with case numbers up to 600 [11–13]. The aim of this multicenter study was to determine the prevalence of GADA, IA2A, and IAA in a large cohort of T1D children of Han Chinese ethnicity.

## RESULTS

### Prevalence of autoantibodies

The mean (± SD) age at diagnosis was 8.3 ± 4.2 years. Of the 750 T1D patients, 66.3% had GADA, 65.3% had IA2A, and 35.7% had IAA (Table 1). Regarding the distribution of positivity of autoantibodies, 48.8% had both GADA and IA2A, 17.5% had only GADA, 16.5% had only IA2A, and 17.2% had no autoantibodies (Table 2).

**Table 1:** Demography and autoantibodies of patients with type 1 diabetes mellitus

Cases	Male: Female (%:%)	AAD (yr) Mean ± SD (range)	Duration (yr) Mean ± SD (range)	GADA Pos/Total (%)	IA2A Pos/Total (%)	IAA Pos/Total (%)
750	357:393 (47.6:52.4)^*^	8.3 ± 4.2 (0.5–19.0)	1.4 ± 3.0 (0.0–18.5)	497/750 (66.3)	490/750 (65.3)	99/277 (35.7)

Abbreviations: AAD, age at diagnosis; Duration, duration of type 1 diabetes; GADA, Glutamic acid decarboxylase 65 autoantibody; IA2A, insulinoma-associated protein 2 autoantibody; IAA, Insulin autoantibody; Pos, positive; SD, standard deviation.

**Table 2:** Positive rate of autoantibodies in 750 patients with type 1 diabetes

GADA	IA2A	Cases	%
+	+	366	48.8
+	–	131	17.5
–	+	124	16.5
–	–	129	17.2

Abbreviations: GADA, Glutamic acid decarboxylase 65 autoantibody; IA2A, insulinoma-associated protein 2 autoantibody.

### Disease duration and prevalence of autoantibodies

Disease duration was 1.4 ± 3.0 years and ranged 0.0 –18.5 years (Table 1). The prevalence of GADA, IA2A, and either GADA or IA2A significantly decreased along T1D duration (Table 3). The positivity of either GADA or IA2A was 89.4% within the first year of disease and decreased to 36.7% after 9 years (*P* = 1.22 × 10^–20^). Logistic regression analysis also confirmed a significant decrement of positivity of GADA, IA2A, and either GADA or IA2A along disease duration (ORs were 0.83, 0.76, and 0.77, respectively; *P* values were all < 0.0001) (Table 6).

**Table 3:** Prevalence of autoantibodies according to duration of type 1 diabetes

Positive/Total (%)						
Duration (yr)	<1.0	1.0 – <3.0	3.0 – <6.0	6.0 – <9.0	≥9.0	*P*^*a*^
GADA	413/569 (72.6)	27/48 (56.2)	36/67 (53.7)	12/36 (33.3)	9/30 (30.0)	1.22 × 10^–20^
IA2A	418/569 (73.5)	31/48 (64.5)	24/67 (35.8)	11/36 (30.6)	6/30 (20.0)	2.34 × 10^–18^
GADA or IA2A	509/569 (89.4)	38/48 (79.2)	44/67 (65.7)	19/36 (52.8)	11/30 (36.7)	1.22 × 10^–20^

*^a^χ*^2^ test.

Abbreviations: GADA, Glutamic acid decarboxylase 65 autoantibody; IA2A, insulinoma-associated protein 2 autoantibody.

**Table 6:** Logistic regression OR for sex, disease duration and age at diagnosis in 750 patients with type 1 diabetes

	β	SE	OR (95% CI)	*P*
**GADA**				
Females	0.57	0.16	1.77 (1.28–2.44)	<0.0005
Disease duration (year)	–0.19	0.03	0.83 (0.78–0.88)	<0.0001
Age at diagnosis (year)	–0.03	0.02	1.03 (0.99–1.07)	0.20
**IA2A**				
Females	0.014	0.16	0.99 (0.71–1.36)	0.93
Disease duration (year)	–0.27	0.03	0.76 (0.72–0.82)	<0.0001
Age at diagnosis (year)	–0.03	0.02	0.97 (0.93–1.01)	0.10
**GADA or IA2A**				
Females	0.57	0.21	1.76 (1.16–2.68)	0.008
Disease duration (year)	–0.26	0.03	0.77 (0.73–0.82)	<0.0001
Age at diagnosis (year)	–0.04	0.03	0.97 (0.93–1.03)	0.32

Abbreviations: GADA, Glutamic acid decarboxylase 65 autoantibody; IA2A, insulinoma-associated protein 2 autoantibody; OR, odds ratio; SE, standard error.

### Sex and prevalence of autoantibodies

The percentage of sex among T1D patients was not different (*P* = 0.35) (Table 1). But female patients had significantly higher prevalence of GADA within one year of diagnosis (77.0% vs. 67.4%, *P* = 0.01), during follow-up (55.7% vs. 37.6%, *P* = 0.015), and through disease duration (72.3% vs. 59.7%, *P* = 0.00027) compared with male patients (Table 4). However, there was no significant difference in the positivity of IA2A between female and male patients (*P* > 0.69). Logistic regression analysis confirmed that female patients had significantly higher positivity of GADA than male patients (OR, 1.77; 95% CI, 1.28–2.44; *P* < 0.0005) (Table 6).

**Table 4:** Comparison in positivity of GADA and IA2A between male and female patients with type 1 diabetes

Positive/Total (%)									
Dis. dur.	<1 year (569 patients)			≥1 years (181 patients)			All (750 patients)		
Antibody	GADA	IA2A	Either	GADA	IA2A	Either	GADA	IA2A	Either
Male	178/264 (67.4)	196/264 (74.2)	231/264 (87.5)	35/93 (37.6)	38/93 (40.9)	52/93 (55.9)	213/357 (59.7)	234/357 65.5)	288/357 (80.7)
Female	235/305 (77.0)	222/305 (72.8)	278/305 (91.1)	49/88 (55.7)	34/88 (38.6)	60/88 (68.2)	284/393 (72.3)	256/393 (65.1)	338/393 (86.0)
*P*^*a*^	0.01	0.69	0.15	0.015	0.76	0.09	0.00027	0.91	0.05

*^a^χ*^2^ test (comparison between males and females within each age groups).

Abbreviations: Dis. dur., Disease duration; GADA, Glutamic acid decarboxylase 65 autoantibody; IA2A, insulinoma-associated protein 2 autoantibody.

### Age at diagnosis and prevalence of autoantibodies

To minimize the confounding of disease duration, samples drawn within half a year of diagnosis were analyzed for the prevalence of autoantibodies at different ages at diagnosis. Positivity of GADA, IA2A, and either GADA or IA2A significantly differed among groups of age at diagnosis (*P* = 0.046, *P* = 0.015, and *P* = 0.011, respectively) (Table 5). The groups of patients diagnosed before 12 years of age had similar positive rates of around 90% (mean 92.2%, Table 7) with the highest rate of 96.4% in the age group of 0.5 – <3.0 years for either GADA or IA2A. However, patients diagnosed at ages 12 or above had a significantly lower positive rate of 81.6% (*P* = 0.011) (Tables 5 and 7). Logistic regression analysis confirmed that the positivity of IA2A and either GADA or IA2A significantly decreased with the increment of age at diagnosis (OR = 0.93, *P* = 0.002 and OR = 0.91, *P* = 0.007, respectively) (Table 8).

**Table 5:** Positivity of autoantibodies within 0.5 year of diagnosis stratified by age at diagnosis

Antibody			Positive/Total (%)				
	All	0.5 – <3.0	3.0 – <6.0	6.0 – <9.0	9.0 – <12.0	12.0–19.0	*P*^*a*^
GADA	397/536 (74.1)	44/55 (80.0)	61/97 (62.9)	105/134 (78.4)	97/125 (77.6)	90/125 (72.0)	0.046
IA2A	397/536 (74.1)	47/55 (85.4)	76/97 (78.4)	104/134 (77.6)	90/125 (72.0)	80/125 (64.0)	0.015
GADA or IA2A	481/536 (89.7)	53/55 (96.4)	88/97 (90.7)	124/134 (92.5)	114/125 (91.2)	102/125 (81.6)	0.011

*^a^χ*^2^ test (5 × 2 contingency table).

Abbreviations: GADA, Glutamic acid decarboxylase 65 kD (GAD) autoantibody; IA2A, insulinoma-associated protein 2 autoantibody.

**Table 7:** Positivity of autoantibodies within 0.5 year of diagnosis stratified by age at diagnosis

Antibody	Positive/Total (%)		
	0.5 – <12.0	12.0–19.0	*P*^*a*^
GADA	307/411 (74.7)	90/125 (72.0)	0.54
IA2A	308/411 (74.9)	80/125 (64.0)	0.016
GADA or IA2	379/411 (92.2)	102/125 (81.6)	0.0006

*^a^χ*^2^ test (2 × 2 contingency table).

Abbreviations: GADA, Glutamic acid decarboxylase 65 autoantibody; IA2A, insulinoma-associated protein 2 autoantibody.

**Table 8:** OR for sex and age at diagnosis on positive autoantibodies within 0.5 year of diagnosis of type 1 diabetes in 536 patients

Antibody	β	SE	OR (95%CI)	*P*
**GADA**				
Females	0.409	0.199	1.50 (1.02–2.22)	0.040
Age at diagnosis (year)	0.008	0.024	1.01 (0.96–1.06)	0.72
**IA2A**				
Females	0.021	0.201	1.02 (0.69–1.51)	0.92
Age at diagnosis (year)	–0.075	0.024	0.93 (0.88–0.97)	0.002
**GADA or IA2A**				
Females	0.47	0.29	1.59 (0.90–2.81)	0.11
Age at diagnosis (year)	–0.095	0.035	0.91 (0.85–0.98)	0.007

Abbreviations: GADA, Glutamic acid decarboxylase 65 autoantibody; IA2A, insulinoma-associated protein 2 autoantibody; OR, odds ratio; SE, standard error.

### Correlation between positivity of GADA and IA2A

The positivity of GADA and IA2A significantly correlated with each other in all T1D patients (Spearman rank correlation coefficient rs = 0.245, *P* = 1.09 × 10^–11^). The correlation was still present even after the data were stratified by sex (rs = 0.293, *P* = 1.69 × 10^–8^ for male patients and rs = 0.203, *P* = 5.13 × 10^–5^ for female patients).

## DISCUSSION

Our study demonstrated that T1D children had a high prevalence (89.4%) of autoantibodies of either GADA or IA2A within the first year of disease duration. We also found the highest positive rate (96.4%) of either GADA or IA2A in T1D diagnosed at the age of 0.5 – <3.0 years. The prevalence of either GADA or IA2A declined with the disease duration and with age at diagnosis. In terms of sex, females had a higher GADA positive rate compared to males. Furthermore, T1D diagnosed before 12 years of age was associated with a higher positive rate of IA2A. There was a weak correlation between GADA positivity and IA2A positivity.

### Disease duration and prevalence of autoantibodies

GADA, IA2A, or both were present in 89.4% of T1D children within one year after diagnosis and their prevalence was around 70% within the first 3 years. Therefore, both GADA and IA2A render the greatest diagnostic value in type 1 diabetes [14].

The dynamics of GADA and IA2A in T1D are complex. The autoantibodies can occur prior to clinical diagnosis and persist years after [15], but they may become undetectable in any period of time [16]. In general, the prevalence declines from the time of diagnosis onwards. Glutamic acid decarboxylase (GAD) and insulinoma-associated protein 2 (IA2) are intracellular antigens in β-cells. In order for β-cell autoantibodies to develop, intracellular autoantigens must be accessible. As a result of cell-mediated autoimmune damage to β-cells, intracellular antigens are released. Consequently, GADA and IA2A develop in response to the released sequestered antigens [17]. As the disease progresses, β-cell mass decreases due to continuous autoimmune destruction resulting in the waning of the autoantigens and thus autoantibodies decline [18]. The gradual decline of the prevalence of GADA and IA2A along the disease duration noted in our study is in accord with previous reports [19].

### Ethnicity and prevalence of autoantibodies

The prevalence of GADA or IA2A varies in different ethnicities. The positivity of GADA is 79% in Germany [20] and Belgium [21], and that of IA2A is 69% in Sweden [14]. On the contrary in Asia, the prevalence of autoantibodies is lower with a GADA positivity of only 44.3% in Singapore [22] and an IA2A positivity of 25.8% in China [13]. However, GADA is positive in 73% and IA2A in 76% of T1D patients within 3 weeks of disease duration from Taiwan [23]. Our results were consistent with the findings of the latter study.

### Differences in GADA prevalence between male and female patients

In our cohort of T1D patients there was no difference in number by sex, however, GADA prevalence was more prominent in females. A female predominance in GADA prevalence remains controversial [18, 24]. Many studies reported GADA was more frequent in females [18, 22, 25, 26], but other studies found no sex difference [19, 24, 27–29]. The female predilection to autoimmunity has been observed in many autoimmune diseases [30, 31]. However, this sex predilection varies among various disorders. The female prevalence of autoimmune diseases ranges from around 90% to 50%, in descending order from Sjögren syndrome, systemic lupus erythematosus, autoimmune thyroid disease, myasthenia gravis, rheumatoid arthritis, multiple sclerosis, ulcerative colitis to T1D [32, 33]. Nonradiographic axial spondyloarthritis and ankylosing spondylitis are respectively the early and late stages of the spectrum of axial spondyloarthritis [34]. Although ankylosing spondylitis is less prevalent in females [35], nonradiographic axial spondyloarthritis is more frequent in females with a prevalence of about 65% [36]. These suggest the importance of female sex in the pathogenesis of autoimmune diseases.

Sex hormones, sex chromosomes, and fetal microchimerism are implicated in this dichotomy [33, 37–39]. Estrogen enhances the Th2 pathway, which activates B lymphocytes to produce antibodies [40, 41]. For example, in systemic lupus erythematosus, estrogen increases the production of anti-DNA antibodies [42, 43]. A female has two X chromosomes. Either the paternal or maternal X chromosome is randomly inactivated, mostly at a ratio of 50:50 [44]. This inactivation may be skewed or incomplete, thereby rendering females more prone to autoimmunity. Fetal microchimerism, the passage of fetal cells into the mother’s circulation and tissues via the placenta, has been found to be associated with autoantibody positivity and autoimmune diseases [45]. It might induce a graft-vs-host reaction or a host-vs-graft reaction [45]. However, many studies have failed to identify the association [46, 47]. Further research using standardized, sensitive, and validated methods is emphasized [46].

### Age at diagnosis and prevalence of autoantibodies

We detected no correlation between the prevalence of GADA and age at diagnosis. Neither did other researchers in studies on Asians [19, 23] and those on Caucasians [24]. However, some researchers found the prevalence of GADA was positively correlated with age at diagnosis in Caucasians [18, 21]. The prevalence of IA2A in our study was age-dependent and negatively correlated with age at diagnosis. This is in accordance with studies on Caucasians [21] except one report [18]. Another study found no correlation in Asians [19]. Type 1B diabetes which is not immune-mediated is more common in patients of African or Asian ancestry [1, 48]. The discrepancy in the correlation between age at diagnosis and prevalence of autoantibodies could be due to the difference in ethnicity, time of sampling since diagnosis, and prevalence of type 1B diabetes in individual populations.

### Correlation between GADA and IA2A

Among our patients, 48.8% had both GADA and IA2A. The positivity of the two autoantibodies correlated with each other, although the correlation coefficient was around 0.2 and regarded as weak [49]. In a previous study from Northern Taiwan, only 15.5% of the 174 T1D patients had both GADA and IA2A [50], which is significantly lower than ours (*P* = 1.24 × 10^–15^). The disease duration in that study was 4.7 years, which was significantly longer than 1.4 years of disease duration in our study (*P* < 0.001). This suggests that our results may more accurately reflex the true correlation between GADA and IA2A.

### Limitations

Although the study consists of the largest cohort of T1D patients in Asians, IAA was only tested in a limited number of patients. Further studies should focus on the measurement of other autoantibodies including ZnT8A particularly in those without GADA and IA2A.

## CONCLUSIONS

There were detectable autoantibodies up to 89.7% in T1D patients within half a year after diagnosis. The prevalence of GADA and IA2A declined with TID disease duration. The prevalence of GADA was higher in female patients than in male ones. IA2A positivity was age-dependent and negatively correlated with age at diagnosis. There was a weak correlation between GADA and IA2A positivity.

## MATERIALS AND METHODS

### Patients

The subjects were recruited from two medical centers, MacKay Memorial Hospital and Chang Gung Memorial Hospital. There were 750 patients (357 males, 393 females) diagnosed with T1D (Table 1). Type 1 diabetes (T1D) was diagnosed on the basis of clinical manifestations and laboratory evidence [51, 52]. Patients had a fasting plasma glucose level ≥7 mmol/l (126 mg/dl) at least 2 times, an HbA1c level of ≥6.5%, or a random glucose level ≥11.1 mmol/l (200 mg/dl) with diabetic symptoms, and at least one of autoantibodies to islet cell antigens, glutamic acid decarboxylase (GAD) and insulinoma-associated protein 2 (IA2) [39, 53] or C-peptide level <0.7 mmol/l (2.1 ng/ml) at random or <1.1 mmol/l (3.3 ng/ml) at the peak by a glucagon test [54].

Their mean (± SD) age at diagnosis was 8.3 ± 4.2 (range, 0.5–19.0) years. The Hospital Institutional Review Board approved this study, and all subjects and their parents or guardians gave written informed consent to participate in this study.

### Methods

Sera of patients were collected and stored in aliquot at –75° C until analysis. Disease duration was defined as the time from diagnosis of T1D till blood sampling. The specimens for IAA were collected within 2 weeks of disease duration. GADA, IA2A, and IAA were measured by radioimmunoassay using ^125^I labeled human GAD-65, human recombinant IA2, and human insulin (^125^I-Tyr-A14-insulin), respectively (CIS Bio International, France). The cut-off level for positivity was set at the 99.5th percentile of control populations. The positivity was >1 U/mL for GADA, >1 U/mL for IA2A, and >5.5% for IAA. The intra-assay coefficient of variation (CV) was 3.6% for GADA, 2.6% for IA2A, and 2.4% for IAA. The inter-assay CV was 6.9% for GADA, 4.3% for IA2A, and 3.1% for IAA.

### Statistical analysis

Numerical data are shown as mean ± standard deviation (SD) and categorical data are shown as numbers and percentages. Patients were stratified into groups by disease duration or age at diagnosis. The duration or age equal to, greater than the initial value, and less than the end value were included in that group. Differences in prevalence of autoantibodies between disease-duration groups, between males and females, or between age-at-diagnosis groups were assessed using *χ*^2^ test. The effect of sex, disease duration, or age at diagnosis on the prevalence of autoantibodies was also assessed with binary logistic regression. The Spearman rank correlation coefficient was assessed between GADA and IA2A. All analyses were performed using PASW Statistics 18 (IBM Corporation, USA). A *P* value of < 0.05 (2-tailed) was considered statistically significant.
